# P-2252. Bloodstream Infections in Heart Transplant Recipients Following the 2018 OPTN Heart Allocation Policy Change

**DOI:** 10.1093/ofid/ofae631.2405

**Published:** 2025-01-29

**Authors:** Alyssa K Mezochow, Marcus R Pereira, Juan Ortega-Legaspi, Ebbing Lautenbach

**Affiliations:** Hospital of the University of Pennsylvania, Philadelphia, Pennsylvania; NYP-Columbia University Irving Medical Center, New York, NY; University of Pennsylvania, Philadelphia, Pennsylvania; University of Pennsylvania, Philadelphia, Pennsylvania

## Abstract

**Background:**

In 2018, the Organ Procurement and Transplant Network (OPTN) changed the United States’ heart allocation policy to prioritize candidates requiring temporary mechanical circulatory support (tMCS), resulting in more orthotopic heart transplant (OHT) candidates transplanted on tMCS. Use of tMCS is associated with nosocomial infections in non-transplant patients, including bloodstream infections (BSI). However, this is not well studied in the OHT population. This study aimed to explore changes in BSI rate in OHT recipients following the 2018 policy change.

**Figure:**
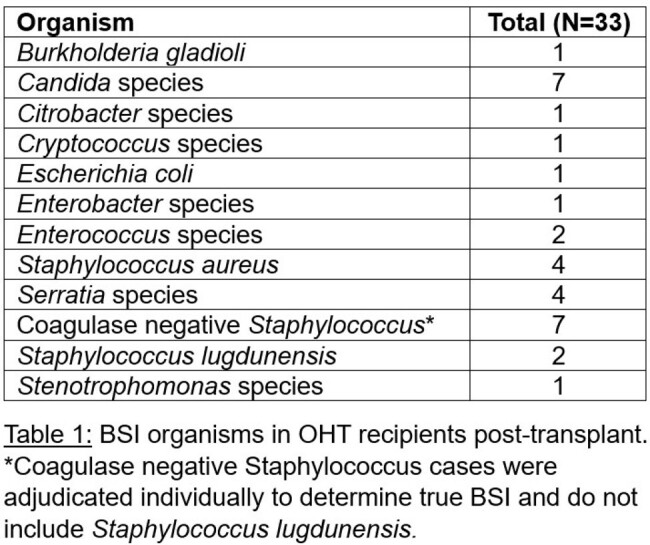

**Methods:**

This was a single center retrospective cohort study of OHT recipients at a large quaternary care medical center from 1/1/2015-12/31/2022. Recipients were followed for 90 days after OHT for initial BSI. BSI organisms were categorized based on microbiology and commensal organisms were classified per the Centers for Disease Control and Prevention definition.

**Figure:**
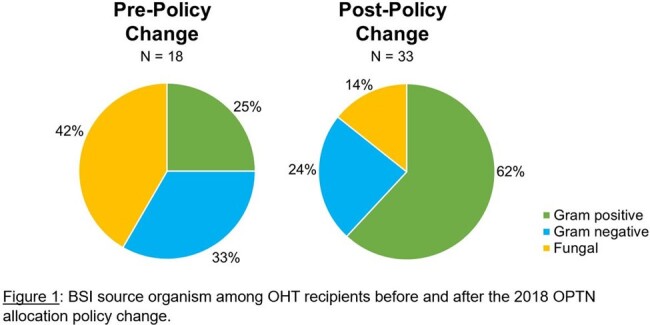

**Results:**

There were 441 OHTs during the study period with 232 (52.6%) OHTs occurring prior to the policy change in 2018. Demographics of recipients did not significantly differ over this period. Overall, 7.5% (N=33) of recipients in the cohort developed a BSI post-transplant. BSI in the cohort were due to 12 unique organisms (Table 1). Nearly half were Gram-positive (48.5%), while Gram-negative and fungal organisms made up a smaller proportion (27.3% vs 24.2%). Recipients in the cohort post policy change had twice the rate of BSI as compared to those transplanted pre-change (10.1% vs 5.2%, P=0.039). Median time to BSI post-transplant in the cohort following the policy change was 11.5 days (IQR 8-18.5), which was similar to prior to the change (14 days, IQR 8-30.5). Fungal infections were more common in the earlier cohort, while Gram-positive infections were more common in the later cohort (Figure 1). Infections with commensal organisms also occurred more frequently in the later cohort (25% vs 48%).

**Conclusion:**

Post-transplant BSI were more common among OHT recipients transplanted after the 2018 allocation policy change. Gram-positive BSI were most common with commensal organisms causing more BSI in the later cohort. Further multivariable analysis and multicenter studies are needed to explore the possible reasons for the increasing BSI rate seen following the policy change.

**Disclosures:**

Marcus R. Pereira, MD, MPH, FAST, Clirnet: Advisor/Consultant|Synklino: Advisor/Consultant|Takeda: Advisor/Consultant Juan Ortega-Legaspi, MD, PhD, Impulse Dynamics: one time paid speaker

